# Equivalent Cellular and Humoral Immunity to Varicella Zoster Virus in Patients With Inflammatory Bowel Disease and Healthy Older Adults for Whom Immunization Is Recommended

**DOI:** 10.14309/ctg.0000000000000446

**Published:** 2022-01-19

**Authors:** Freddy Caldera, Arnold Wald, Sumona Saha, Ryan Smith, Sue McCrone, Francis A. Farraye, Mary S. Hayney

**Affiliations:** 1 Department of Medicine, Division of Gastroenterology and Hepatology, University of Wisconsin—Madison, School of Medicine & Public Health, Madison, Wisconsin, USA;; 2 Department of Medicine, Division of Internal Medicine, University of Wisconsin Madison, School of Medicine & Public Health, Madison, Wisconsin, USA;; 3 School of Pharmacy, University of Wisconsin—Madison, School of Medicine & Public Health, Madison, Wisconsin, USA;; 4 Inflammatory Bowel Disease Center, Division of Gastroenterology and Hepatology, Mayo Clinic, Jacksonville, Florida, USA.

## Abstract

**INTRODUCTION:**

Patients with inflammatory bowel disease (IBD) are at an increased risk of herpes zoster (HZ). HZ is caused by reactivation of the varicella zoster virus (VZV) and is prevented by strong VZV-specific cell-mediated immunity. The aim of our study was to evaluate whether patients with IBD had lower or equivalent protection compared with healthy controls (HCs) at age 50 years and older.

**METHODS:**

We performed a cross-sectional study at a single academic center and evaluated cellular and humoral immunity to VZV in patients with IBD at age 35–49 years vs HCs aged 50–59 years. All patients with IBD were on stable medication regimens for at least 3 months. VZV-specific cell-mediated immunity was measured *via* ELISPOT, and humoral immunity was measured *via* a quantitative VZV antibody enzyme-linked immunosorbent assay assay.

**RESULTS:**

Seventy-seven patients with IBD and 12 HCs were enrolled in the study. There was no significant difference in ELISPOT counts between patients with IBD and HCs (*P* = 0.54). In addition, there was also no significant difference between ELISPOT counts in immunosuppressed patients with IBD (N = 45) and HCs (*P* = 0.32). We also found no correlations between ELISPOT counts and age (Spearman rho 0.014; *P* = 0.90). Patients with IBD had similar IgG VZV antibody levels (median 19 mIU/mL; range 0.5–218) compared with HCs (median 23.5 mIU/mL (range 4–34); *P* = 0.54).

**DISCUSSION:**

Young patients with IBD have equivalent cellular and humoral immunity to VZV as healthy older adults in whom HZ immunization is recommended.

## INTRODUCTION

Patients with inflammatory bowel disease (IBD) are at a 2-fold increased risk of herpes zoster (HZ), independent of whether they are on immunosuppressive therapy, compared with the general population (1). Those on thiopurines, a combination of thiopurines and an antitumor necrosis factor (anti-TNF)-alpha agent, or corticosteroids are at an increased risk of HZ compared with those on aminosalycilates (1,2). Ustekinumab is a human monoclonal antibody to interleukin-12/23 approved to treat IBD but has not been shown to be associated with an increased risk of HZ in pooled phase 2 and 3 studies (3). Tofacitinib, a nonselective Janus kinase inhibitor, was approved in 2018 to treat ulcerative colitis (UC) and is associated with a higher risk of HZ compared with conventional agents (4).

HZ is caused by reactivation of latent varicella zoster virus (VZV) due to age-related waning of VZV-specific cell-mediated immunity (CMI) (5). It is known that VZV-specific CMI, rather than VZV-specific antibodies (humoral immunity), plays a critical role in maintaining latency of VZV and preventing HZ (5). Interferon (IFN)-γ enzyme-linked immunospot (ELISPOT) is the preferred method to measure VZV-specific CMI because it directly measures the number of T cells secreting IFN-γ after stimulation with VZV antigen (6–8). Large epidemiologic studies in healthy individuals aged 50 years or older demonstrated that those with lower VZV-specific CMI were at a higher risk of developing HZ or postherpetic neuralgia (7,9,10). Furthermore, VZV-specific CMI significantly decreases with increasing age, and when subjects were divided into age groups, there were significant differences in IFN-γ spot-forming cells between 50-year-old and 60-year-old healthy adults (7,10). VZV-specific CMI was lower in an immunosuppressed cohort of patients with systemic lupus erythematous or granulomatosis with polyangiitis when compared with healthy controls (HCs) (8). This finding is consistent with population-based studies that have shown that patients with autoimmune conditions are at an increased risk of HZ (11). Administrative and health claim–based studies suggest that patients with IBD are at an increased risk of HZ compared with controls (1,5,12). They have found that 40-year-old patients with IBD have a greater risk of HZ than do healthy 50-year-old individuals, an age when HZ immunization is typically recommended (5,13,14). The recombinant subunit HZ vaccine (RZV) is an inactivated highly immunogenic vaccine that in 2 phase 3 clinical trials, the vaccine demonstrated 97% efficacy in individuals aged 50 years and older and 89% efficacy in individuals aged 70 years and older (15,16). The RZV is a 2-dose series currently licensed for adults aged 50 years and older in Canada, Japan, Australia, and China but recently was given approval for adults aged 18 years and older who are or will be at increased risk of HZ due to immunosuppression caused by known disease or therapy in the United States and European Union (5,13,17).

In the United States, licensure of the vaccine does not guarantee insurance coverage of the vaccine until the Advisory Committee on Immunization Practice (ACIP) makes a recommendation. The Affordable Care Act states that ACIP recommendations for immunization are required to be covered without cost sharing (18). Thus, until their recommendation, a significant portion of patients may not be eligible for the RZV series due to cost. This is a significant issue for patients with IBD on immune-modifying therapy who are at a higher risk of HZ (4,19). The aim of our pilot study was to evaluate whether young patients with IBD (age 35–49 years) had lower or equivalent protection against HZ compared with HCs aged 50 years and older.

## METHODS

### Study design

This cross-sectional pilot study compared quantitative VZV antibody and ELISPOT counts in patients with IBD and HCs who presented for routine clinical care at the University of Wisconsin Digestive Health Center (Madison, WI) between December 2017 and May 2019. The inclusion criteria for both cohorts required a history of chicken pox, no history of any zoster vaccine or HZ, and positive VZV qualitative antibody. Zoster vaccine history was verified in the Wisconsin Immunization Registry, an Internet immunization information system tracking immunization histories of Wisconsin residents, which is linked to the electronic health record (20). The HCs cohort consisted of 12 individuals aged 50 years or older. VZV-specific CMI in HCs has been well characterized in previous studies, and the goal of our pilot study was to evaluate VZV-specific CMI in patients with IBD (21).

The inclusion criteria for the IBD cohort were age 35–49 years, having an established diagnosis of IBD, and on stable medical treatment for at least 3 months. The age of 35 years and older was chosen because we wanted to include only individuals who had varicella infection (chicken pox) and not those immunized with varicella vaccine, which started in 1995 and was routinely used for children through age 12 years (22). The varicella vaccine contains an attenuated VZV, and this probably makes them less susceptible to HZ compared with those with a history of varicella infection (23,24). Thus, we excluded patients who received a varicella vaccine from both cohorts. The IBD cohort needed to be on stable medical treatment for at least 3 months, and they were divided into the following groups: (i) mesalamine or no IBD therapy; (ii) azathioprine ≥ 2 mg/kg or 6-MP ≥ 1 mg/kg, which are standard dosing for treatment of IBD (25); (iii) anti-TNF-α monotherapy; (iv) anti-TNF in combination with immune modulator (thiopurine or methotrexate); (v) vedolizumab monotherapy or in combination with immune modulator; and (vi) corticosteroids and anti-TNF monotherapy or combination therapy. Patients could be enrolled if they had an active disease or were in clinical remission. A single blood sample was conducted to measure CMI to VZV using ELISPOT. A qualitative VZV antibody assay was conducted at the time of enrollment in the study to assure that all participants were immune to VZV. Lymphocytes and plasma were frozen until assayed.

### Immune measures

ELISPOT is an enzyme-linked assay for detecting and enumerating lymphocytes that produce cytokines in response to an antigen. Cytokine ELISPOT sets for IFN-γ (BD Biosciences Pharmingen, San Diego, CA) were used according to the manufacturer's instructions. Two hundred thousand lymphocytes were incubated with VZV antigen (BioRad; Hercules, CA) (8,21). Cytokine-producing cells were counted using AID imaging system (Strassberg, Germany). VZV-specific cell response was calculated by subtracting the number of cells that produced IFN-γ in the no-antigen control well. Varicella zoster IgG in plasma was measured by enzyme-linked immunosorbent assay (ELISA) (IBD, Minneapolis, MN) and adjusted to the World Health Organization First International Standard for varicella zoster immunoglobulin (1987) (National Institute for Biological Standards and Control, Hertfordshire UK).

### Data analysis

The primary outcome of the study was to compare VZV-specific CMI *via* ELISPOT between patients with IBD and HCs. The secondary outcomes were as follows: (i) evaluating VZV-specific CMI between types of IBD and immunosuppressed patients with IBD and those with IBD on nonimmunosuppressing regimens (ii) comparing VZV-specific antibodies between patients with IBD and HCs; (iii) evaluating correlation between age and VZV-specific CMI; and (iv) correlation between VZV-specific CMI and VZV-specific antibodies.

The VZV-specific immune responses measured *via* ELISPOT were compared among groups using the Kruskal-Wallis tests and between groups using the Mann-Whitney *U* tests. A Spearman correlation was used to determine whether relationships existed between responder cell frequency and antibody concentration or age. Our sample sizes conferred an 87% power to detect a difference of 20 responder cells in HCs to 10 responder cells in patients with IBD (8).

### Ethical considerations

The study protocol was approved by the University of Wisconsin-Madison Health Sciences Institutional Review Board, and each individual gave written informed consent before participating in the study.

## RESULTS

A total of 77 patients with IBD and 12 HCs were enrolled in the study. By design, the HCs were older than the patients with IBD, but the groups were comparable regarding other characteristics, with the exception that the nonimmunosuppressed group had more patients with UC compared with the other groups (Table 1). Forty-six (60%) patients with IBD experienced Crohn's disease, and the remainder of the cohort experienced UC.

**Table 1.** Demographics of study participants

	Control N = 12	5 ASA or no treatment N = 24	Thiopurine^a^ N = 12	Anti-TNF^b^, N = 12	Combination regimens^c^ N = 13	Vedolizumab^d^ N = 10	Prednisone regimens^e^ N = 6	*P*-value^f^
Age, yr (median [range])	51.5 [50–59]	44 [32–49]	40 [35–49]	41 [35–46]	42 [35–48]	43 [37–47]	43 [35–48]	<0.001
Male (%)	7 (58)	10 (41.6)	6 (50.0)	6 (50.0)	7 (53.8)	4 (40.0)	4 (66.7)	0.91
White (%)	10 (92)	23 (95.8)	12 (100)	11 (91.7)	13 (100)	9 (90.0)	6 (100)	0.51
Type of IBD								
Ulcerative colitis (%)		16 (66.7)	4 (33.3)	3 (25.0)	3 (23.1)	4 (40.0)	1 (16.7)	0.045
Active disease (PM ≥ 2) (%)		2 (12.5)	1 (25.0)	0	1 (33.3)	1 (25.0)	1 (100)	0.31
Crohn's disease (%)		8 (33.3)	8 (66.7)	9 (75.0)	10 (76.9)	6 (60.0)	5 (83.3)	0.045
Active disease (HBI ≥5) (%)		2 (25.0)	2 (25.0)	1	1 (10.0)	1 (16.7)	3 (60.0)	0.25
Length of disease, mo (median [range])		120 [4–288]	99 [11–432]	164 [12–324]	99 [22–432]	146 [32–336]	253 [20–319]	0.33
Current therapy								
Aminosalicylates (%)		19 (79.2)	4 (33.3)	1 (8.3)	0	0	2 (33.3)	
Azathioprine (%)		0	11 (91.7)	0	9 (69.2)	1 (10.0)	1 (16.7)	
6-mercaptopurine (%)		0	1 (8.3)	0	3 (23.1)	0	0	
Anti-TNF (%)		0	0	12 (100)	13 (100)	0	6 (100)	
Methotrexate (%)		0	0	0	1 (7.7)	0	2 (33.3)	
Vedolizumab (%)		0	0	0	0	10 (100)	0	
Average daily thiopurine dose (median [range])		—	150 [50–200]	—	125 [50–250]	^g^	^h^	
Length of current therapy, mo (median [range])		58 [3–181]	41 [5–219]	31 [3–112]	35 [4–102]	11 [5–33]	4 [1–6]	
Previous therapy								
Budesonide in the past yr (%)		1 (4.2)	1 (8.3)	4 (33.3)	3 (23.1)	3 (30.0)	4 (66.7)	
Prednisone in the past yr (%)		4 (16.7)	2 (16.7)	2 (16.7)	2 (15.4)	3 (30.0)	6 (100)	
Anti-TNF (%)		8 (12.5)	3 (8.3)	12 (41.7)	9 (30.8)	8 (30.0)	7 (50.0)	
Thiopurine (%)		2 (4.2)	0	8 (66.7)	5 (15.4)	4 (30.0)	5 (50.0)	
Methotrexate (%)		3 (12.5)	0	4 (33.3)	0	3 (30.0)	1 (16.7)	
Vedolizumab (%)		0	0	1 (8.3)	1 (7.7)	0	1 (16.7)	
Ustekinumab (%)		0	0	0	0	0	1 (16.7)	
5 ASA (%)		10 (37.5)	5 (25.0)	9 (50.0)	7 (38.5)	8 (80.0)	7 (83.3)	

No statistical comparisons were performed for IBD treatments among groups because the groups were different by study design.

ASA, aminosalicylates; Anti-TNF, antitumor necrosis factor; CD, Crohn's disease; HBI, Harvey Bradshaw Index; IBD, inflammatory bowel disease; IQR, interquartile range; PM, Partial Mayo.

a Thiopurines = azathioprine ≥ 2 mg/kg or 6-MP ≥ 1 mg/kg.

b Anti-TNF = anti-TNF monotherapy.

c Anti-TNF in combination with immune modulator (thiopurine or methotrexate).

d Vedolizumab monotherapy or in combination with immune modulator.

e On anti-TNF maintenance therapy as combination or monotherapy along with at least 10 mg of prednisone.

f The Kruskal-Wallis tests.

g Two individuals were taking 50 or 100 mg of azathioprine daily.

h One individual was taking 100 mg of azathioprine daily; 2 others were on 12.5 and 25 mg of methotrexate.

### VZV-Specific CMI in patients with IBD

**Figure 1.**
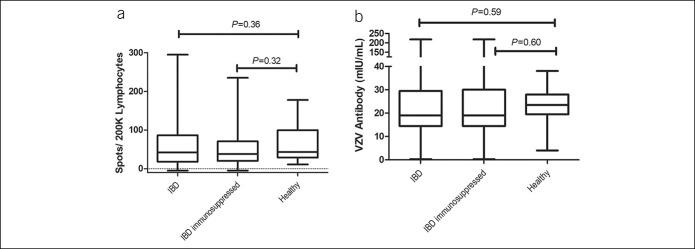
Varicella zoster virus (VZV) immune measures by all patients with inflammatory bowel disease (IBD) and healthy controls (HCs). Each boxplot shows the median, 25th and 75th percentiles, and range. (**a**) Cell-mediated immunity (CMI) to VZV comparisons with HCs and patients with IBD and immunosuppressed patients with IBD (includes patients on thiopurines, anti-TNF monotherapy, combination therapy, and corticosteroids and 2 patients on vedolizumab and azathioprine) with HCs (Mann-Whitney *U* tests). (**b**) VZV antibody concentrations were also compared for patients with IBD and HCs. Again, no statistically significant differences between patients with IBD and HCs were measured (Mann-Whitney *U* tests). We found no difference in either immune measure compared with healthy individuals aged 50 years and older for whom zoster immunization is recommended.

**Figure 2.**
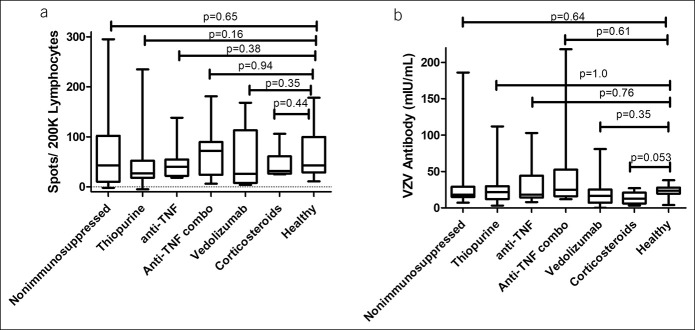
Varicella zoster virus (VZV) immune measures by treatment group. Each boxplot shows the median, 25th and 75th percentiles, and range. (**a**) Cell-mediated immunity (CMI) to VZV comparisons with HCs is not statistically significantly different for any inflammatory bowel disease (IBD) treatment group (Mann-Whitney *U* tests). (**b**) VZV antibody concentrations were also compared for each IBD treatment groups with HCs. Again, no statistically significant differences between IBD treatment group and HCs were measured (Mann-Whitney *U* tests). We found no difference in either immune measure compared with healthy individuals aged 50 years and older for whom zoster immunization is recommended.

All patients with IBD and HCs were able to respond to VZV stimulation. The median number of IFN-γ spot-forming cells per 2 × 10^5^ PBMC in response to VZV stimulation was 43 (range 11–178) among HCs and 76 (range 5–295) among patients with IBD (*P* = 0.36) (Figure 1). There was also no significant difference between IFN-γ spot number in immunosuppressed patients with IBD and HCs (*P* = 0.32) (Figure 1). In addition, among the individuals with IBD, no difference was found in patients with UC (median 42 spots; range 5–295) and those with Crohn's disease (median 34 spots; range 1–239; *P* = 0.84) or between those who were treated with a nonimmunosuppressing regimen, those in Group A and vedolizumab monotherapy, (median 42 spots; range 5–295), and those who were on an immunosuppressing regimen (median 43 spots; range 11–178; *P* = 1.0). Furthermore, there was no significant difference in the median number of IFN-γ spots among the IBD groups categorized by treatment regimen (Figure 2). We also found no correlations between IFN-γ spots and age (Spearman rho 0.014; *P* = 0.90).

### VZV quantitative antibodies

All patients with IBD and HCs had measurable antibodies per inclusion criteria. Patients with IBD had similar IgG VZV antibody levels (median 19 mIU/mL range 0.5–218) compared with HCs (median 23.5 mIU/mL [range 4–38]; *P* = 0.59), as measured with the quantitative IgG ELISA and adjusted to the World Health Organization standard. Among immunosuppressed patients with IBD, IgG VZV antibody levels (median 19 mIU/mL [range 0.5–218]; *P* = 0.60) did not differ significantly from IgG VZV antibody levels in nonimmunosuppressed patients (Figure 1). In addition, there was no difference in IgG VZV antibody levels across all groups (*P* = 0.42; Kruskal-Wallis test). We also found no difference in VZV antibody concentrations between those with UC (median 17 mIU/mL; range 0.5–218) and Crohn's disease (median 19; range 3–198; *P* = 0.35). They also did not correlate with VZV-specific cell number (Spearman rho 0.063; *P* = 0.55).

## DISCUSSION

In this study, patients with IBD with a laboratory-confirmed history of varicella infection showed equivalent cellular and humoral immunity to VZV as healthy older adults in whom HZ immunization is recommended. This is the first study to evaluate the protection against HZ by measuring VZV-specific CMI in patients with IBD. It provides biological support for the findings in health claim–based studies that patients with IBD younger than 50 years have at least a similar risk of HZ compared with healthy individuals aged at least 50 years (1,2). We did not find a difference between the groups, as was seen by Rodaan et al. (8) who found that VZV-specific CMI was lower in those with an autoimmune disorder compared with HCs, but their cohorts were of similar age and may account for differences in VZV-specific CMI because it decreases with age. Our study was designed to determine whether young patients with IBD with previous VZV infection had equivalent or lower VZV-specific CMI compared with older HCs and not compared with age-matched controls. We chose to evaluate HCs in whom HZ immunization is recommended because if we found equivalent or lower VZV specific CMI, this would provide biological evidence for their known increased risk of HZ and would suggest that younger patients with IBD may benefit from early HZ immunization. The immunosuppressive therapies used to treat IBD are known to be independent risk factors for HZ, and a recent report suggested that HZ may be an adverse event of fecal microbiota transplantation, a common treatment for patients with IBD with recurrence of *Clostridium difficile* (1,2,26). Thus, patients with IBD would benefit from getting immunized with the RZV series, which has been associated with decreased risk of HZ (27).

Sustained VZV-specific humoral immunity has been evaluated in adult patients with IBD previously using commercial ELISA assays (28,29). These studies have found that most of the adults maintain immunity to VZV (28). Our study did not find a difference in IgG VZV antibody concentrations in patients with IBD compared with HCs. This is similar to other studies in HCs showing that IgG VZV antibody concentrations do not decline with age and are not useful in predicting reactivation of VZV (5). VZV-specific antibodies are accurate at predicting a previous VZV infection, protection of infection, but have no role in predicting the development of HZ or postherpetic neuralgia. VZV-specific CMI plays a critical role in prevention reactivation of VZV because declining VZV-specific CMI contributes to HZ risk, and boosting of VZV-specific CMI *via* immunization is protective against HZ (7,9,10,30).

Immunosuppressive therapies used to treat IBD are associated with an increased risk of HZ. Anti-TNF therapy, corticosteroids, and thiopurines have all been found to be independently associated with HZ, and those on combination therapy have the highest risk of HZ (1,2). The mechanism of action of thiopurines and anti-TNF therapy may help to explain the increased risk of HZ. Thiopurines inhibit proliferation of T and B lymphocytes and reduce the number of cytotoxic T cells (31,32). TNF plays a critical role in controlling viral infections by recruiting and activating macrophages, T cells, and antigen-presenting cells (33). TNF also reduces VZV replication and VZV antigen expression *in vitro* (34). Depletion of TNF by treatment with anti-TNF therapy may facilitate the reactivation of VZV (33). Similar to Rodann et al. (8), we did not find a difference in VZV-specific CMI between immunosuppressed patients and those nonimmunosuppressed in our study, but our study was not powered to be able to distinguish a difference.

The RZV series is currently recommended for use in immunocompetent adults aged 50 years and older in the Canada, European Union, Japan, Australia, and China and individuals aged 18 years and older in the United States at an increased risk of HZ (5,13,17). In the United States, the RZV is the preferred vaccine of the ACIP, which is composed of medical and public health experts who developed recommendations on the use of vaccine in the United States, but they have yet to make any specific recommendations regarding immunosuppressed populations (5). The Food and Drug Administration approved the RZV series in those aged 18 years and older who are at increased risk of HZ and it is anticipated the ACIP will provide more detailed recommendation of which patients 18 years and older should receive the RZV series.

The RZV series has been evaluated in immunosuppressed adults 18 years or older with hematological malignancies and after kidney transplant and found to be safe and immunogenic, but there are no published studies evaluating the immunogenicity in patients with IBD (35,36). In a small case series, RZV was found to be safe in patients with IBD (37). However, our pilot study found that patients with IBD with a history of varicella infection had equivalent VZV-specific CMI compared with HCs. Future studies are needed to determine which patients with IBD with a previous varicella infection aged younger than 50 years would benefit from early HZ immunization. Future studies should focus on evaluating the impact that systemic immunosuppressive agents have on VZV-specific CMI to determine whether certain agents significantly lower protection against HZ would be helpful. Population-based studies could also evaluate whether lower VZV-specific CMI is associated a higher risk of developing HZ or postherpetic neuralgia in younger patients with IBD. The most important future study is a randomized controlled trial to measure the efficacy of RZV in patients with IBD. These studies would be helpful to inform gastroenterology preventive care guidelines in North America, Europe, Asia, and other regions to make evidence-based recommendations for RZV use in patients with IBD (14,38,39).

Our study has several strengths and some limitations. Our cross-sectional single-center study was adequately powered to detect a difference between patients with IBD and HCs. We were able to evaluate sustained protection against reactivation of VZV using a gold standard approach to measure VZV-specific CMI. In addition, we were able to confirm previous VZV infection in all patients, which would affect VZV-specific CMI. Our pilot study was limited due to a being a single-center study, small sample size among the different treatment groups, and mostly White population limiting generalizability. We also did not evaluate the effect of tofacitinib on VZV-specific CMI or use age-matched HCs. Despite these limitations, this study has contributed to our knowledge about humoral and cellular immunity to VZV in patients with IBD.

In conclusion, our study found that patients with IBD with previous varicella infection had equivalent VZV-specific CMI compared with HCs for whom the zoster vaccine is recommended. Future studies are needed to determine which patients with IBD younger than age 50 years would benefit from early immunization.

## CONFLICTS OF INTEREST

**Guarantor of the article:** Freddy Caldera, DO, MS.

**Specific author contributions:** F.C.: study concept and design, acquisition of data, analysis and interpretation of data, drafting of the manuscript, critical review of the manuscript for important intellectual content, and statistical analysis. A.W., S.S., and F.A.F.: critical review of the manuscript for important intellectual content. R.S.: acquisition of data and critical review of the manuscript for important intellectual content. S.M.: analysis and interpretation of data and critical review of the manuscript for important intellectual content. M.S.H.: study concept and design, acquisition of data, analysis and interpretation of data, critical review of the manuscript for important intellectual content, and statistical analysis.

**Financial support:** The project described was supported by an American College of Gastroenterology pilot award and University of Wisconsin Clinical and Translational Science Award (CTSA) program, through the NIH National Center for Advancing Translational Sciences (NCATS), grant UL1TR002373. The content is solely the responsibility of the authors and does not necessarily represent the official views of the NIH.

**Potential competing interest:** F.C. has been a consultant for Takeda Pharmaceuticals, Celgene Pharmaceuticals, and Arena Pharmaceuticals and received research support from Takeda Pharmaceuticals and Sanofi for work unrelated to the topic of this manuscript.

**Study Highlights**

**WHAT IS KNOWN**

✓ Patients with inflammatory bowel disease (IBD) are at an increased risk of herpes zoster (HZ).
✓ Many patients are not eligible to be vaccinated against HZ.

**WHAT IS NEW HERE**

✓ Varicella zoster virus (VZV)-specific cell-mediated immunity (CMI) plays a critical role in preventing HZ.
✓ Patients with IBD with previous VZV infection had preserved CMI to VZV stimulation.
✓ Young patients with IBD have equivalent VZV-specific CMI as healthy older adults in whom HZ immunization is recommended.
